# Effects of cholesterol on bilayers with various degrees of unsaturation of their phospholipid tails under mechanical stress

**DOI:** 10.1039/d0ra00624f

**Published:** 2020-03-17

**Authors:** Dongyu Lyu, Lei Zhang, Yong Zhang

**Affiliations:** School of Physics, Sun Yat-Sen University No. 135 Xingang Xi Road Guangzhou 510275 China zhlei28@mail.sysu.edu.cn zhyong9@mail.sysu.edu.cn

## Abstract

Cholesterol is one of the essential components of the cell membrane. It has a significant influence on various mechanical properties of biomembranes, such as fluidity and elasticity, which have attracted much attention. It is also well known that the concentration of cholesterol affects the mechanical strength of cell membranes. In this paper, we aim to explore the influence of the degree of unsaturation of phospholipid tails on the concentration-effect of cholesterol. Three different phospholipids (DPPC, DIPC and DAPC) were selected as the respective main components of the bilayers and several concentrations of cholesterol were also added to the systems. Our coarse-grained molecular dynamics simulations show that as the cholesterol concentration increases, the saturated phospholipid bilayer is first strengthened, by increasing the rupture tension from 68.9 to 110 mN m^−1^, and then weakened. The non-monotonic concentration-effect gradually decreases as the degree of unsaturation of the phospholipid tails increases, and in particular, the mechanical strength of the DAPC bilayer hardly changes. The results suggest that cholesterol does not influence a bilayer composed of highly unsaturated phospholipids. Furthermore, lateral density distributions reveal that the distribution of cholesterol in the bilayer is related to the carbon tail unsaturation of the phospholipids.

## Abstract 2

The concentration-effect of cholesterol on the mechanical strength of biomembranes weakens as the degree of unsaturation of the phospholipid tails increases.

## Graphical abstract

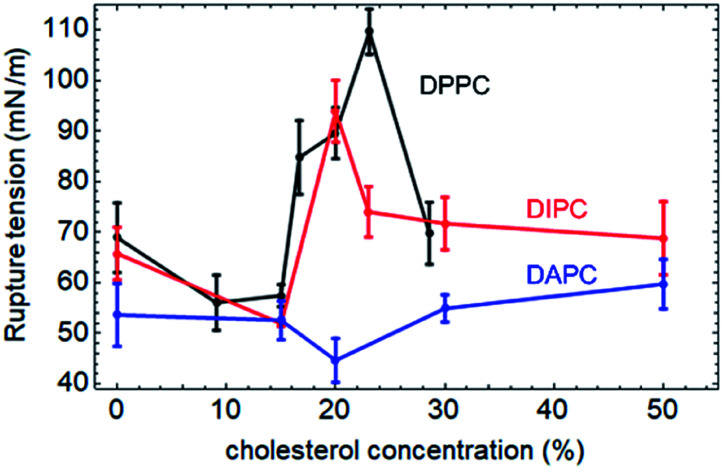

## Introduction

A cell membrane is a fundamental element of a living cell.^1^ It acts as a highly selective barrier between the interior and exterior of a cell and guarantees the relative stability of the intracellular environment so that various biochemical reactions can be carried out in an orderly way.^2^ The composition of a biomembrane is complicated, and a pure phospholipid bilayer is usually employed as a simplified model of a biomembrane in simulation studies.^3–8^

Mechanical stress plays an essential role in the growth, proliferation, differentiation, and apoptosis of cells.^9,10^ Plenty of biomechanical experiments have indicated that a certain level of mechanical stress affects the shapes and structures of living cells.^11–14^ It also regulates the functions and biological activities of cells by affecting the morphologies of membrane proteins in a normal physiological environment.^15^ However, on the membrane, excessive stress leads to the formation of pores, damages the continuity of the structure and, *in vitro*, causes the damage or even the death of the cell.^16–20^

It is well known that the mechanical properties of a lipid membrane are determined by its composition. For example, Venable *et al.* evaluated the mechanical properties of twelve different lipid bilayers and calculated their bending moduli.^6^

Cholesterol is one of the crucial components of biomembranes, and its concentration in cell membranes varies greatly in different types of cells.^1^ It has been revealed that the concentration of cholesterol has a significant influence on the physical properties of biomembranes, such as fluidity,^21,22^ permeability,^23,24^ viscoelasticity,^25^ and even their transportation properties.^26^ Some experiments have shown the effect of cholesterol concentration on the mechanical properties of phospholipid bilayers.^27–30^ Rawicz *et al.* suggested that the strengths of stearoyloleoyl phosphatidylcholine (SOPC) and dioleoyl phosphatidylcholine (DOPC) bilayers would increase significantly with the incorporation of cholesterol. For example, an addition of 50% cholesterol increased the rupture tension of a SOPC bilayer from 12 to 26 mN m^−1^.^27^ Pan *et al.* found out that up to 40% cholesterol did not affect the bending modulus of DOPC bilayers, although it did have a stiffening effect on membranes composed of phospholipids with two saturated chains.^29^ Rajkumar *et al.* revealed that the correct membrane cholesterol levels are crucial to the physical state of the cell. Furthermore, excess cholesterol causes significant mechanical remodeling of the membrane-cytoskeletal structure by triggering molecular signaling in endothelial cells.^30^

However, the pore formation and the rupturing of lipid bilayers are usually very fast events, which makes it difficult to observe them directly in experiments.^31^ Molecular dynamics (MD) simulation is a promising method to solve this problem. By using MD simulation, researchers can not only reproduce molecular scale phenomena in the bilayer but also analyze the physical quantities that are difficult to measure experimentally. Furthermore, a coarse-grained (CG) model allows the simulation of a larger system size and longer time scales for at least one order of magnitude than that in the all-atom MD simulation.

Most of the previous studies have focused on saturated phospholipid bilayers, but in recent years, more and more attention has been paid to their unsaturated phospholipid counterparts.^32–34^ Nanda *et al.* presented experimental measurements and analysis of the dynamics and phase behaviour of saturated dimyristoylphosphatidylcholine (DMPC) and unsaturated DOPC bilayers.^32^ Ermilova *et al.* carried out a series of MD simulations to study the different positions and orientations of the cholesterol inside bilayers composed of phospholipids with various degrees of unsaturation.^33^ Leonov *et al.* discussed the cholesterol concentration dependence of the Raman peak parameters of three phospholipid bilayers.^34^

In the present paper, we aim to explore the effects of cholesterol concentration on the mechanical strength of various phospholipid bilayers with different degrees of unsaturation on the tails. Our results indicate that the addition of cholesterol first strengthened a dipalmitoylphosphatidylcholine (DPPC) bilayer by increasing the rupture tension, then as the concentration kept increasing, the rupture tension decreased. However, this effect decreased with increasing degrees of unsaturation of the phospholipid tail chains. And, the effect of cholesterol was neglectable in a bilayer comprising diarachidonylphosphatidylcholine (DAPC), the carbon tails of which are highly unsaturated.

## Materials and methods

The Martini CG force field was employed in the following CG-MD simulations. It has been widely used in many CG-MD simulations,^35,36^ especially in the field of lipid membranes and proteins.^37^ Also, plenty of molecular level biological phenomena have been reproduced using this model, including the phase transition in phospholipid bilayers^4,38^ and the interaction between phospholipids and nanoparticles.^3^

### 1 Simulation details

**Fig. 1:**
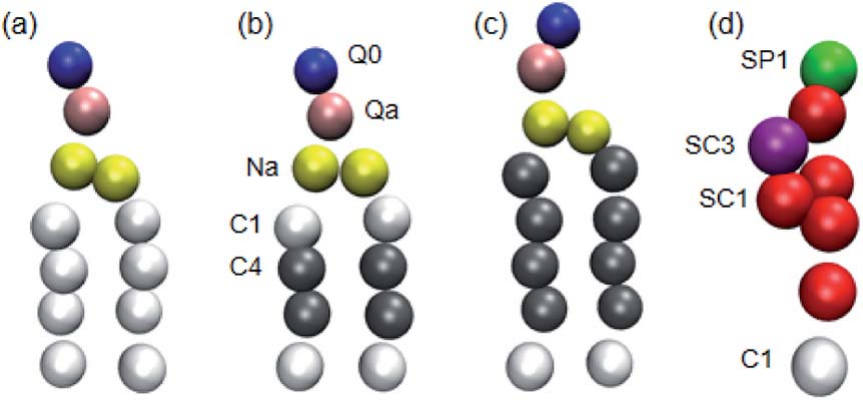
Martini CG models of (a) DPPC, (b) DIPC, (c) DAPC and (d) cholesterol molecules. The different types of Martini CG beads are shown in different colours. In particular, the saturated and unsaturated carbon beads are in white and grey, respectively.

Three phospholipids, DPPC, dilinoleoylphosphatidylcholine (DIPC) and DAPC (the CG models of which are shown in Fig. 1), were chosen for the simulations. These phospholipids have the same headgroup, but significantly different carbon tails, especially in terms of their degree of unsaturation. The initial coordinate files of the bilayers with cholesterol were obtained using a bilayer formation program provided by Wassenaar's group.^39^ A phospholipid–cholesterol bilayer was placed in a cubic simulation box with a side length of 13 nm. This bilayer contains 510 molecules in total (in some cases 512 molecules) and was fully hydrated with around 14 000 CG water beads. We changed the ratio of the cholesterol and phospholipid molecules, but the total number of these two molecules was almost maintained.

**Fig. 2:**
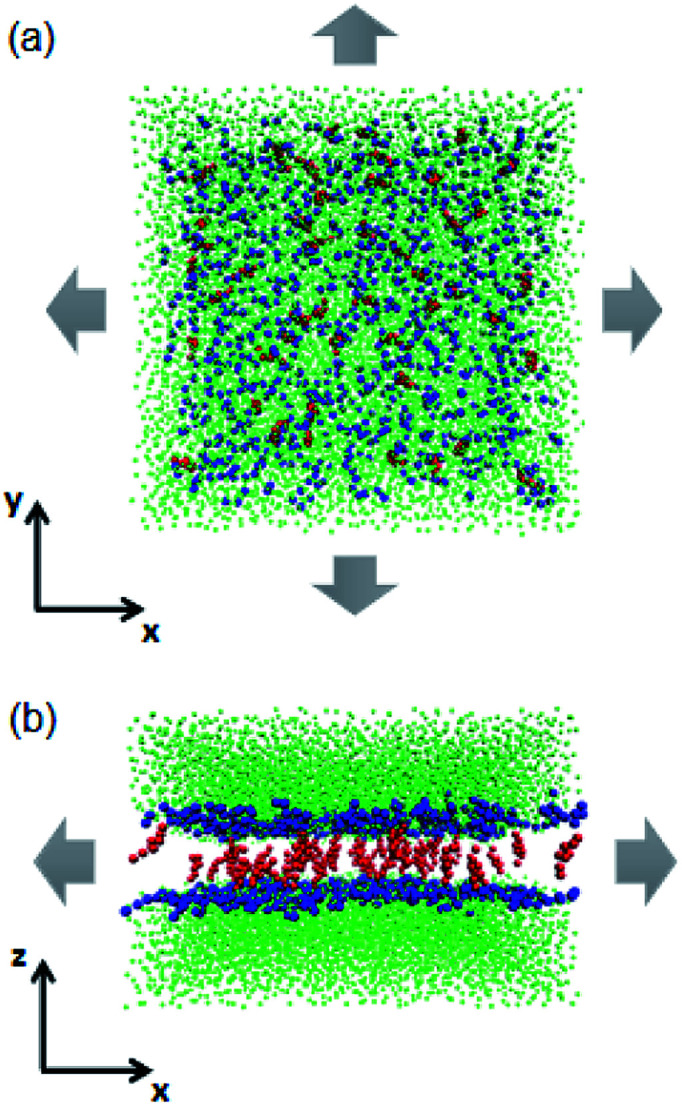
Schematic illustrations of the tense lipid bilayer system. (a) Top view. (b) Side view. Headgroups of the phospholipids are represented by blue beads, cholesterol molecules are in red and water beads are in green. The grey arrows indicate the mechanical stress which was applied in the lateral direction. The phospholipid tails are not shown for clarity.

In experiments, the stretching and rupturing of phospholipid bilayers are usually implemented using a micropipette with a suction pressure applied to vesicles.^27,28^ In the present work, a semi-isotropic pressure coupling method was used. It allows the normal and lateral pressure to change independently for the bilayers to implement the mechanical stress (as shown in Fig. 2). Each system was equilibrated at 325 K, which makes sure that the bilayers are in the fluid phase state,^38^ under ambient pressure (zero surface tension) for 1 μs first. Then, a higher lateral pressure (from −50 to −80 bar, where the negative sign indicates a tensile force) was applied to the simulation box until the bilayers broke.

All simulations were performed using the Gromacs software, version 5.1.1.^40–42^ The time step of the simulations was set to 20 fs. Periodic boundary conditions were applied, and the temperature and pressure were achieved using the Berendsen algorithm.^43^ The coupling constant of the temperature was 0.1 ps, and 0.2 ps was used for the constant of the pressure. Lennard-Jones (L-J) and electrostatic potentials were cut off at 1.1 nm. Visual Molecular Dynamics (VMD) software, version 1.9.4,^44^ was applied for the visualization of the simulation results.

### 2 Calculation of surface tension

The surface tension *γ* is determined by the difference of the normal and lateral pressure in the simulation box,^3,38^*γ* = *L*_*z*_(*P*_N_ − *P*_L_),where *L*_*z*_ is the critical normal length of the box when the membrane ruptures, and *P*_N_ and *P*_L_ are the normal and lateral pressures of the box, respectively, which can be calculated from the normal (*P*_*zz*_) and lateral (*P*_*xx*_ and *P*_*yy*_) components of the stress tensor,*P*_N_ = *P*_*zz*_,
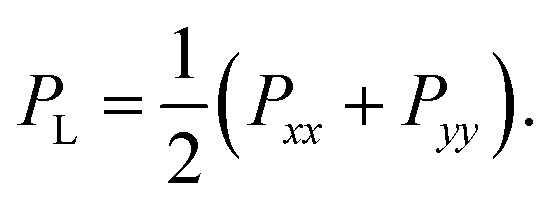


The rupture tension of the lipid bilayer, which is the critical surface tension at the moment of rupture, was applied as an index for evaluating the mechanical strength of the bilayer. The higher the rupture tension, the stronger the bilayer.

### 3 Calculation of the order parameter

The order parameter of phospholipid tails *S*_*z*_ is calculated using the following equation,^45^
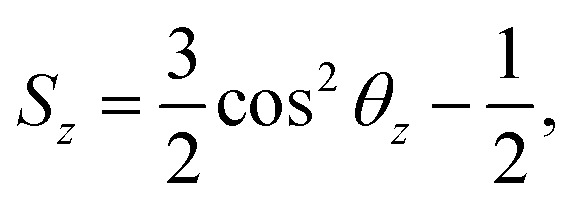
where *θ*_*z*_ is the angle between the *z*-axis of the system and the vector from the Martini CG beads *C*_*n*−1_ to *C*_*n*+1_ for beads *C*_*n*_. The order parameter indicates the orientation of carbon chains and it varies from 1 to −1/2.

## Results and discussion

### 1 Rupture tension

**Fig. 3:**
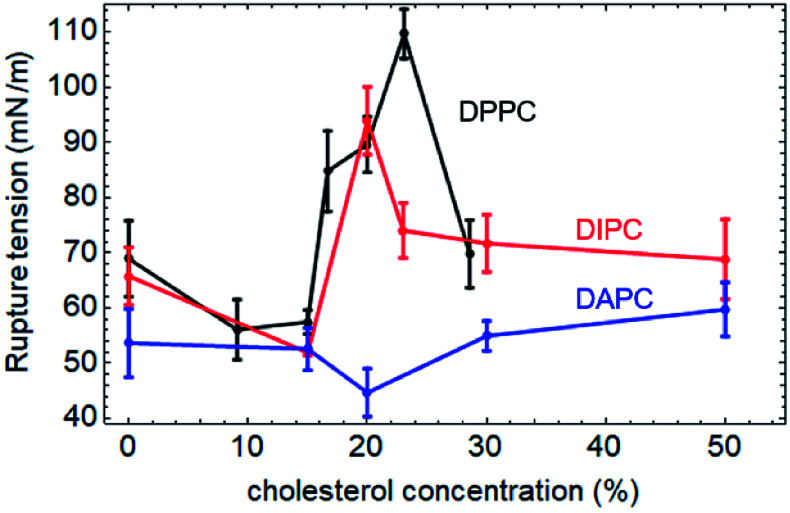
The rupture tensions of the lipid bilayers as a function of the concentration of cholesterol. In the phospholipids we chose, the carbon tails of the DAPC molecule have the highest degree of unsaturation, while the tails of the DPPC molecule have the lowest.

The rupture tensions of the three types of phospholipid bilayers containing different concentrations of cholesterol are shown in Fig. 3. As for the pure phospholipid bilayers, the greater the degree of unsaturation of the phospholipid tails, the higher the rupture tension. Also, the concentration-effect of cholesterol on the mechanical strength of these membranes is significantly different.

Both tail chains of DPPC are fully saturated. The rupture tension of the DPPC bilayer increases gradually up to 110 mN m^−1^ with an increase in the cholesterol concentration to 23%. However, when the concentration of cholesterol keeps increasing to 29%, the mechanical strength of the bilayer decreases to 69.8 mN m^−1^.

The DIPC bilayer shows a similar tendency, with a lower peak value of 93.9 mN m^−1^ when the cholesterol concentration is at 20%.

However, in the case of DAPC, which is a type of phospholipid with highly unsaturated carbon tails, the concentration-effect of cholesterol is negligible. The incorporation of cholesterol molecules hardly affects the mechanical strength of the DAPC bilayer. The maximum change in the rupture tension of the bilayer containing an amount of cholesterol is less than 17%, compared with that of the pure one.

The all-atom simulations carried out by Shigematsu *et al.* showed that the critical areal strain of the DPPC bilayer initially increased with the addition of cholesterol molecules, peaked at 40%, and then decreased,^46^ which indicated the same tendency as our results. However, it should be noticed that the strain and stress are conceptually different, although there is a correlation between them. Both can indicate the mechanical strength of the membrane. This explains why the cholesterol concentrations at the peak in the present work are not exactly the same as the results reported by Shigematsu *et al.*

Rawicz *et al.* applied the different phospholipid bilayers DOPC and SOPC to study the influence of the cholesterol concentration on the mechanical strength of the phospholipid bilayers experimentally.^27^ Both of the two tails of DOPC are unsaturated, while, one of the tails of SOPC is saturated, and the other is unsaturated. An addition of 50% cholesterol increased the rupture tension of the DOPC and SOPC bilayers by 90% (from 10 to 19 mN m^−1^) and 117% (from 12 to 26 mN m^−1^), respectively. This indicates that the greater the degree of unsaturation, the lower the impact of the cholesterol, which supports our results.

### 2 Pore formation

When high mechanical stress is applied to the bilayer, the penetration of water molecules occurs more frequently than at low mechanical stress. A typical pore formation process of the membrane has been observed in our simulations, which is consistent with the previous studies.^47–49^

**Fig. 4:**
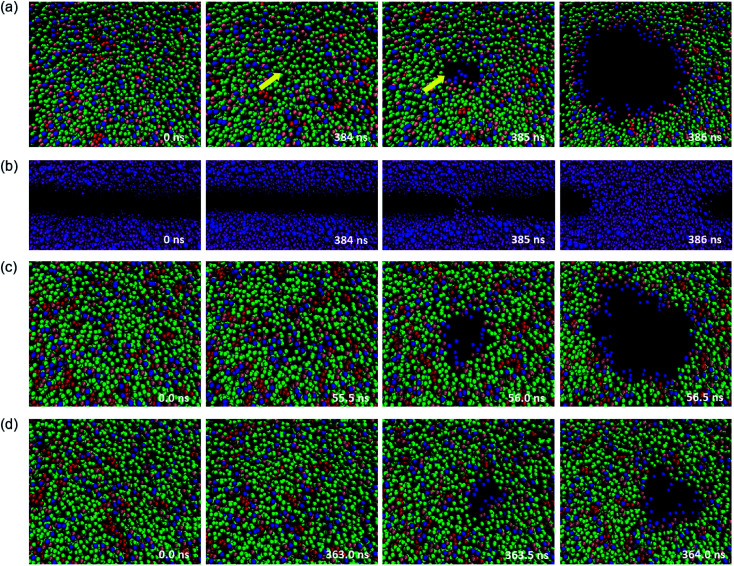
Typical process of pore formation in a phospholipid bilayer under high mechanical stress. (a) A top view of the DPPC bilayer with 9.1% cholesterol molecules. Headgroups, backbone and carbon tails of phospholipids are represented by blue, pink and green beads, respectively, cholesterol molecules are shown in red. The yellow arrows point to the pore formation area. (b) Side view. Headgroups of tails are in blue and water beads in purple. The other molecules are not shown for clarity. The complete process of water molecules penetrating the bilayer can be observed. (c) DIPC–cholesterol bilayer. (d) DAPC–cholesterol bilayer.

The structure of a few typical snapshots during the pore formation and the rupture of the phospholipid–cholesterol membranes are shown in Fig. 4. At first, a small hydrophobic pore appears in the membrane and the phospholipid headgroups are significantly inclined due to thermal and mechanical fluctuations. The top view shows that the phospholipid headgroups are scattered, and only the carbon tail chains are observed in the pore area, as shown in Fig. 4(a). Then, the water beads quickly fill the hydrophobic pore, followed by the complete formation of the water bridge, as shown in Fig. 4(b). This indicates the formation of a hydrophobic pore in the interior of the bilayer. Afterwards, the heads of the local phospholipids rearrange at the edge of the pore and towards the center of the pore, which indicates the changing of the pore from hydrophobic to hydrophilic.

The driving force of the pore formation on the surface of the biomembranes comes from the competition between the reduction in the interfacial energy of the system and the additional energy required to form the pores. The interfacial energy reduced by the formation of the pore plays a more critical role when a tensile force is applied.

We also paid attention to the distribution of cholesterol in the *x*–*y* plane of the system, and the relationship between cholesterol and pore formation locations. The results show that for a stress-free membrane (zero surface tension), the cholesterol molecules are evenly distributed in the center of the bilayer. Also, rupture only occurs between phospholipids when the cholesterol concentration is at least not over 30%, which is an important phenomenon. This typical membrane surface pore formation process and the location characteristics of cholesterol are independent of the degree of unsaturation of the phospholipid tails.

### 3 Lateral density distributions

**Fig. 5:**
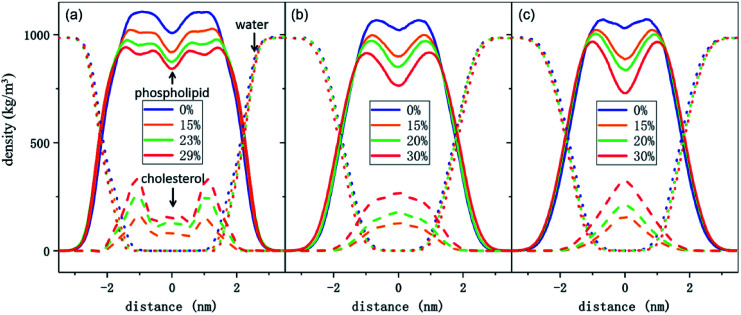
Lateral density distributions for several phospholipid bilayer systems under zero surface tension. (a) DPPC bilayers with different concentrations of cholesterol molecules. The density of phospholipids, cholesterol and water molecules are shown as full, dashed, and dotted lines, respectively. (b) DIPC–cholesterol bilayers. (c) DAPC–cholesterol bilayers. All lines in this figure were obtained after processing.

**Fig. 6:**
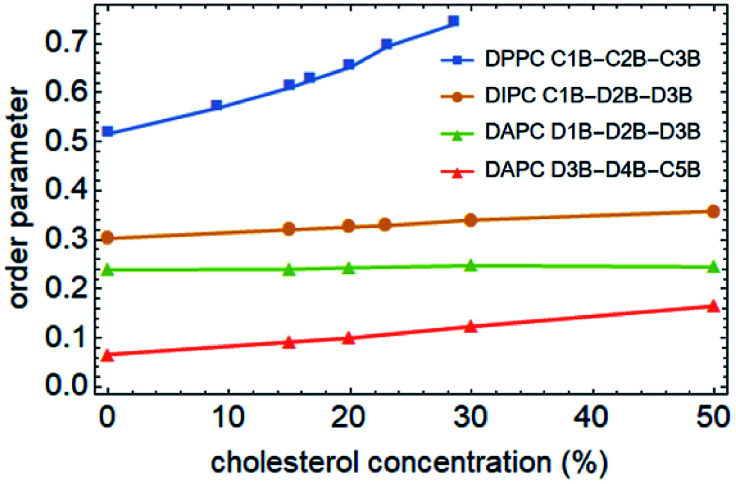
Average order parameters of the phospholipid tails as a function of cholesterol concentration. Only one carbon chain of the two was calculated. The data points of DPPC, DIPC and DAPC are marked using squares, dots and triangles, respectively.

To better understand the cholesterol concentration effect on the mechanical strength of the different phospholipid bilayers, we examined the lateral density distributions extracted from the simulation data, as shown in Fig. 5. One can obtain the thickness of the bilayers by measuring the distance between the two intersection points of the density curves of the phospholipids and water molecules in lateral density distributions. This value is increased by the addition of the cholesterol at zero surface tension and decreased monotonically under the tension just before rupture for all of the phospholipid membranes in this study. The cholesterol molecules occupy a part of the volume in the bilayer and reduce the available space for the phospholipid tails. This led to an increase in the order parameter of the carbon chains, as shown in Fig. 6. An increase in the order parameter leads to a thickening of the membrane.

Furthermore, the results show that the distributions of the cholesterol in these membranes are highly dependent on the degree of unsaturation of the phospholipid carbon tails. For the DPPC bilayer, the lateral density distribution of the cholesterol is a bimodal curve, which shows the same characteristics as DPPC molecules. Cholesterol is a hydrophobic molecule. It has a length comparable to that of the phospholipid tail.^50^ When cholesterol is added into the DPPC bilayer, the similar distributions of the cholesterol and the phospholipid tails cause a strong interaction to occur between them. This helps them to form a uniform hydrophobic barrier and enhance the strength of the membrane.

On the other hand, for the unsaturated phospholipid bilayers, the cholesterol molecules are concentrated in the center of the bilayers, which is a unimodal distribution. Moreover, as observed in lateral density distributions, the density distribution of cholesterol in the center of the DAPC bilayer is sharper than in the DIPC one. This indicates that the higher the degree of unsaturation of the phospholipid tails, the more concentrated the distribution of the cholesterol in the normal center of the bilayer. At the same time, cholesterol molecules occupy a larger volume in the center of the unsaturated phospholipid bilayers, and as a result, the staggered interaction of the two layers of carbon tail chains is weakened. This explains not only the significantly lower density contribution of unsaturated phospholipids in the middle of the bilayer in Fig. 5, but also why the incorporation of cholesterol only affects the order parameters of the phospholipid tails in the interdigitation area between the two layers instead of the part near the headgroup, as the green and red lines show in Fig. 6.

These different cholesterol distribution characteristics signify that cholesterol prefers a saturated bilayer over an unsaturated one.^51^ And, this might result in a difference in the rupture tension between these bilayers, but the relevance of this needs further exploration.

### 4 Discussion

As has been observed, ruptures of membranes only occur between phospholipid molecular pairs at low cholesterol levels, but not around cholesterol molecules. The higher the cholesterol concentration in the system, the smaller the area ratio occupied by phospholipids, and the less likely it is for pores to form. On the other hand, cholesterol aggregation is enhanced with increasing concentration. The area of phospholipid that may form pores is greater, and as a result, the rupture tension decreases. The mutual influence of the two mechanisms makes the membrane rupture tension exhibit a non-monotonic response with the cholesterol concentration.

**Fig. 7:**
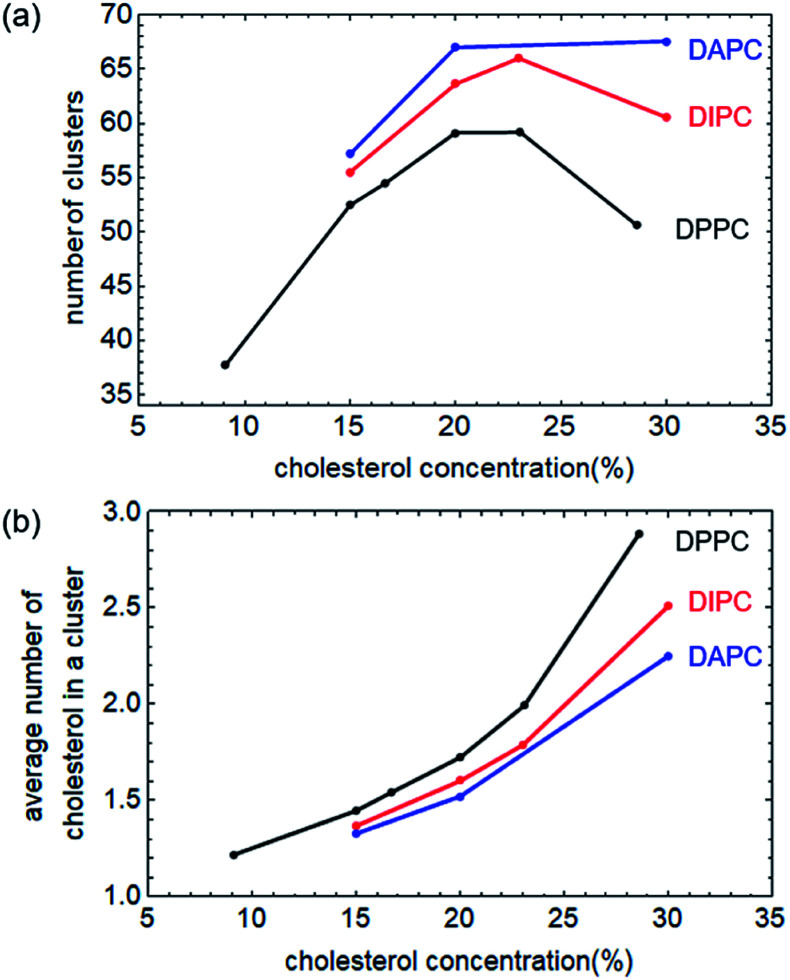
(a) Number of clusters of cholesterol as a function of its concentration. Structures of the last 100 nanoseconds of the equilibrium system were extracted from the trajectory file and analysed. For cholesterol in the DPPC and DIPC bilayers, the number of clusters showed a significant non-monotonic change. In contrast, the number of cholesterol clusters in DAPC is monotonic. (b) The average number of cholesterol molecules in each cluster increases as the cholesterol concentration increases, revealing enhanced cholesterol aggregation.

We counted the number of cholesterol clusters of each system under zero surface tension, as shown in Fig. 7(a). The average size of a cluster, measured by the average number of cholesterol molecules in a cluster, is shown in Fig. 7(b). Our current qualitative explanation aims to clarify that the effect of cholesterol concentration on the rupture tension of bilayers is not monotonic, also the intensity of this effect is highly related to the degree of the unsaturation of the phospholipid tails.

The number of cholesterol clusters in the DPPC bilayers first increases and then decreases with increasing concentration, peaks at 23%, which is approximately consistent with the simulation results shown in Fig. 3. And, as a comparison, the cluster numbers of cholesterol in the unsaturated bilayers do not change much when the concentration is not higher than 30%, which means that the cholesterol concentration has little effect on the rupture tension of the unsaturated phospholipid bilayers.

## Conclusions

In the present work, we investigated the effect of the cholesterol concentration on the mechanical properties of three different bilayers composed of phospholipids with different degrees of unsaturation in the tail chains. Tensile forces were applied in the radial direction of the bilayers, and the critical surface tensions were calculated to characterize the mechanical strengths of the bilayers. The results of the CG-MD simulations suggest that the incorporation of cholesterol strengthens the phospholipid bilayer. Also, there is a specific concentration of cholesterol that maximizes the rupture tension of the bilayer. If the cholesterol concentration continues to increase, the membrane is weakened. According to the simulations, the rupture tension of the DPPC bilayer increased from 68.9 to 110 mN m^−1^, upon an addition of 23% cholesterol to the system. This effect of the cholesterol on the phospholipid bilayers decreases with increasing degree of unsaturation of the phospholipid tails. An addition of 20% cholesterol increases the rupture tension of the DIPC bilayer from 65.8 mN m^−1^ to a maximum value of 93.9 mN m^−1^. The strength of the DAPC bilayer remains almost the same when the concentration of cholesterol is in the range of 0% to 50%.

## Conflicts of interest

There are no conflicts to declare.

## Supplementary Material
